# Implementation of the “l’O à la bouche” Therapeutic Patient Education Program at a French Cancer Center: A Multidisciplinary Approach for Managing Post-Cancer Oral Toxicities

**DOI:** 10.1007/s13187-025-02716-w

**Published:** 2025-09-01

**Authors:** Caroline de Bataille, Nathalie Caunes-Hilary, Aurélie Robello, Sabine Bétancourt, Emmanuelle Vigarios, Delphine Maret

**Affiliations:** 1 Département de Soins de Support, IUCT Oncopole—Institut Claudius Regaud, Toulouse, France; 2 Département Consultations, IUCT Oncopole—Institut Claudius Regaud, Toulouse, France; 3 Département d´Odontologie, Universi té de Toulouse, IUCT-Oncopole, Centre Hospitalier Universitaire, Toulouse, France

**Keywords:** Therapeutic patient education, Head and neck oncology, Oral health, Cancer treatment toxicities, Oral health literacy, Supportive care, Multidisciplinary care, Patient partnership

## Abstract

Head and neck cancer treatments, particularly radiotherapy, cause significant oral toxicities that profoundly impact patients’ quality of life, including salivary modifications, swallowing disorders, and the risk of osteoradionecrosis. While therapeutic patient education (TPE) programs exist for various chronic conditions, there remains a notable gap in specific programs addressing oral complications in head and neck cancer patients. This article describes the development and implementation of the innovative TPE program “l’O à la bouche” at a French cancer center, specifically designed for head and neck cancer patients experiencing post-treatment oral toxicities. A multidisciplinary working group was established, including ENT surgeons, speech-language pathologists, dietitians, radiotherapy technicians, nurses, dentists, psychologists, and patient partners. The program development followed a structured timeline from February 2020 to June 2021. The program comprises four complementary workshops addressing oral physiology modifications, safety and self-monitoring for aspiration risks, practical self-care skills, and psychosocial rehabilitation. The program obtained official authorization in December 2020 and began implementation in June 2021, with stable activity of 12 workshops annually, accommodating 5 to 8 patients per workshop. This initiative addresses a previously unmet national need, as no equivalent TPE programs existed specifically targeting salivary modifications and their oral consequences in head and neck cancer patients. The strong involvement of patient partners throughout all development phases ensures clinical relevance and patient-centered care. This program serves as a model for specialized therapeutic education that can improve patient self-management skills, enhance treatment adherence, and ultimately optimize quality of life through targeted patient education interventions.

## Introduction

Cancer incidence continues to rise globally, requiring a multidisciplinary oncology workforce and innovative care approaches [1, 2]. Beyond survival, patient quality of life has become a major priority, necessitating proactive management of treatment-related adverse effects. Head and neck cancers, in particular, can result in significant oral toxicities, including xerostomia, mucositis, dysphagia, and dysgeusia, profoundly impacting patients’ daily functioning [3, 4]. These challenges underscore a critical need for specialized education and support for this chronic disease.

Therapeutic patient education (TPE), defined by the WHO as helping patients acquire or maintain skills necessary for managing their lives with a chronic disease, offers a promising approach [5, 6]. It marks a shift from a paternalistic care model to person-centered care, recognizing the unique needs of patients, including their health literacy and cultural background [5–7].

In this context, the design and implementation of TPE programs dedicated to oncology-specific needs, particularly oral toxicities, are paramount. The “l’O à la bouche” program at the cancer center (IUCT-Oncopole, Toulouse, France) represents a targeted initiative to address these specific needs.

The objective of this article is to describe the development and structure of this TPE program, positioning it within the broader landscape of international literature regarding oral health literacy and chronic disease management. By detailing this innovative approach, we aim to demonstrate how specialized therapeutic patient education can empower head and neck cancer patients with the knowledge, skills, and confidence needed to effectively self-manage their oral complications and improve their overall quality of life.

## Methods

The “l’O à la bouche” TPE program was initiated by Dr. CdB, a dentist, as part of his University Diploma (DU) in TPE, emphasizing a commitment to integrating dental expertise into patient education. Its development was characterized by a collaborative and multidisciplinary approach, essential for addressing the complexity of oral toxicities in oncology. The co-construction of the program involved a diverse working group, including head and neck surgeons, an onco-sexologist, speech-language pathologists, dietitians, radiologic technologists, a nurse, a nursing assistant in onco-rehabilitation, dental assistants, psychologists, and, most importantly, patient partners. This diversity of expertise reflects the multifaceted nature of oral health challenges in oncology.

The program was developed through nine working group meetings, which enabled the definition of the project and program framework, the construction of the patient competency framework, the development of learning and educational objectives, the creation of tools, and the organization of scheduling and communication. This meticulous work, complemented by four paired meetings for workshop refinement, resulted in obtaining authorization from the Regional Health Agency (ARS) in December 2020, and the launch of workshops in June 2021. The process also involved quadrennial program evaluations and the declaration of associated programs.

The program specifically targets patients with head and neck cancer following completion of radiotherapy who are capable of oral feeding. Its primary objective is to enable them to acquire self-care and safety skills to adapt to their daily lives modified by toxicities. This includes understanding and managing treatment-related changes, enabling them to develop identification and adaptation capabilities to improve their quality of life. The program is divided into four main modules, each addressing key dimensions of patient life:

- My mouth, my saliva, and my swallowing particularities: Covers salivary physiology, identification of different oral structures, an understanding of sequelae following modifications, factors that may increase or decrease salivary modifications, and learning oral cavity self-examination.
- Recognizing and responding to aspiration risk situations: Focuses on safety and self-monitoring by identifying risk situations, learning adaptation strategies for prevention, and training the patient’s support network.
- My nutrition and oral comfort in daily life: Addresses practical oral self-care (such as fluoride application, brushing techniques) and dietary adaptation (food texture, flavors) to restore mealtime pleasure and social life.
- My relationship with others, my emotions, regaining confidence: Aims to rebuild self-esteem, maintain social life, and authorize new experiences.

The program emphasizes practical sessions, with educational tools in constant evolution to adapt to patient needs and treatment advances.

## Discussion

### Comparison with International Literature

The “l’O à la bouche” program is part of a global movement to improve patient care and integrate oral health into general healthcare. Analysis of international sources reveals enriching parallels and points of divergence.

#### Oral Health Literacy (OHL)

A study conducted by Lawler et al. [8] on OHL education in dental hygiene programs revealed that most programs integrate OHL to varying degrees, primarily focusing on communication strategies and identification of patients with low OHL. However, gaps remain regarding formal assessment of literacy levels and teaching all cognitive levels of health literacy [8, 9]. The “l’O à la bouche” program, by focusing on the acquisition of self-care and safety skills, directly contributes to improving patients’ OHL. The fact that patient partners participate in its construction suggests a conscious approach to communication adapted to patient needs. Health literacy is a better predictor of health status than age, income, employment status, or education level.

#### Role of Non-Dental Professionals (NDPs)

Qin et al. [10] demonstrated that, although NDPs (physicians and nurses) have general knowledge of oral health, their specific knowledge for special groups (pregnant women, children) is insufficient. However, these NDPs show great interest in learning and are highly favorable to intersectoral collaboration in oral health [10–12]. This reinforces the relevance of the multidisciplinary character of the “l’O à la bouche” program, where professionals from various disciplines collaborate for integrated education, and underscores the importance of continuing education, such as the 40-h TPE certification completed by dental assistants.

#### Patient Therapeutic Patient Education (TPE) in Chronic Diseases

Regarding carious lesions, Zanini et al. [13] advocate for a paradigm shift toward an educational approach, emphasizing that traditional methods of information dissemination and counseling are often insufficient to promote lasting behavioral changes. They propose applying TPE principles to caries, considered as a chronic disease, by integrating educational diagnosis and behavioral follow-up. The transtheoretical model of behavioral change is suggested to adapt educational strategies to the patient’s psychological stages [14–16]. The “l’O à la bouche” program aligns perfectly with this vision, treating post-cancer oral toxicities as a chronic condition requiring long-term management, patient empowerment, and adoption of new behaviors (e.g., dietary adaptation, specific hygiene). The rarity of specific TPE programs for adults concerning oral modifications, as noted by Zanini et al., positions “l’O à la bouche” as an innovative and essential model in oncology [13].

#### Initiatives in Cancer Education

The importance of oncology interest groups is recognized for stimulating medical students' interest in the specialty and improving overall patient care [17]. These groups facilitate early clinical exposure, research, and empathetic interactions with patients. Although “l’O à la bouche” is a TPE program for patients rather than an interest group for students, its existence and success demonstrate the growing need for specialized education programs within the oncology field, reinforcing the multidisciplinary approach to post-cancer management.

### Challenges and Perspectives

The “l’O à la bouche” program operates within a constantly evolving ecosystem, and its deployment is accompanied by challenges and opportunities. Although operational since 2021, the program faces several challenges: lack of visibility and awareness among healthcare professionals regarding this type of TPE program focused on oral health, reflecting broader gaps in knowledge about oral toxicities of anticancer treatments, as well as logistical difficulties for patients who sometimes travel long distances to attend fixed-date in-person sessions. Additionally, patients are often less informed about available supportive care services, which can limit their engagement with programs that could significantly benefit their treatment experience and quality of life.

#### Continuing Education and Communication

Promoting continuing education for healthcare educators is a priority. These continuous developments and identified challenges are representative of a constant quality improvement approach, essential for adapting TPE programs to the complex and evolving needs of oncology patients. Ultimately, these efforts aim to enhance patient education outcomes by ensuring that educational interventions remain relevant, accessible, and effective in empowering patients to manage their oral health complications.

## Conclusion

The “l’O à la bouche” TPE program at IUCT-Oncopole is an eloquent example of how a multidisciplinary and patient-centered approach can address specific challenges in oncology. By focusing on post-radiotherapy oral toxicities, this program fills an essential gap in comprehensive care for head and neck cancer patients, enabling them to regain control of their oral health and significantly improve their quality of life. The integration of TPE principles, interprofessional collaboration, and patient partner involvement are the pillars of its success, positioning it favorably compared to international initiatives in health literacy and healthcare professional training [10, 13]. The challenges encountered, such as the need to adapt infrastructure and integrate technological tools, are inherent steps in the evolution of any supportive care program. For the future, continued development of new workshops, harmonization of practices within the TPE team, and strengthening links with other institutions and international literature will be crucial for consolidating and disseminating the acquired expertise. The “l’O à la bouche” program constitutes a model that demonstrates how specialized therapeutic patient education can effectively address the complex oral health needs of oncology patients, providing them with essential knowledge and skills for long-term self-management while serving as a blueprint for similar patient education initiatives worldwide.
